# Protocol Development for Point Clouds, Triangulated Meshes and Parametric Model Acquisition and Integration in an HBIM Workflow for Change Control and Management in a UNESCO’s World Heritage Site

**DOI:** 10.3390/s21041083

**Published:** 2021-02-04

**Authors:** Adela Rueda Márquez de la Plata, Pablo Alejandro Cruz Franco, Jesús Cruz Franco, Victor Gibello Bravo

**Affiliations:** 1 Department of Graphic Expression in Architecture, University of Extremadura, 10003 Cáceres, Extremadura, Spain; 2 Department of Construction, University of Extremadura, 10003 Cáceres, Extremadura, Spain; pablocruzfranco@unex.es; 3 Panta Rhei Desarrollo S.L., 10003 Cáceres, Extremadura, Spain; jcruzfranco@gmail.com; 4 Arqveocheck S.L., 06810 Mérida, Extremadura, Spain; arqveocheck@icloud.com or

**Keywords:** modeling, data-fusion, UAV, HBIM, TLS, cultural heritage, cáceres, defensive wall, digital survey

## Abstract

This article illustrates a data acquisition methodological process based on Structure from Motion (SfM) processing confronted with terrestrial laser scanner (TLS) and integrated into a Historic Building Information Model (HBIM) for architectural Heritage’s management. This process was developed for the documentation of Cáceres’ Almohad wall bordering areas, a UNESCO World Heritage Site. The case study’s aim was the analysis, management and control of a large urban area where the urban growth had absorbed the wall, making it physically inaccessible. The methodology applied was the combination of: clouds and meshes obtained by SfM; with images acquired from Unmanned Aerial Vehicle (UAV) and Single Lens Reflex (SLR) and terrestrial photogrammetry; and finally, with clouds obtained by TLS. The outcome was a smart-high-quality three-dimensional study model of the inaccessible urban area. The final result was two-fold. On one side, there was a methodological result, a low cost and accurate smart work procedure to obtain a three-dimensional parametric HBIM model that integrates models obtained by remote sensing. On the other side, a patrimonial result involved the discovery of a XII century wall’s section, that had supposedly been lost, that was hidden among the residential buildings. The article covers the survey campaign carried out by the research team and the techniques applied.

## 1. Introduction

**Figure 1:**
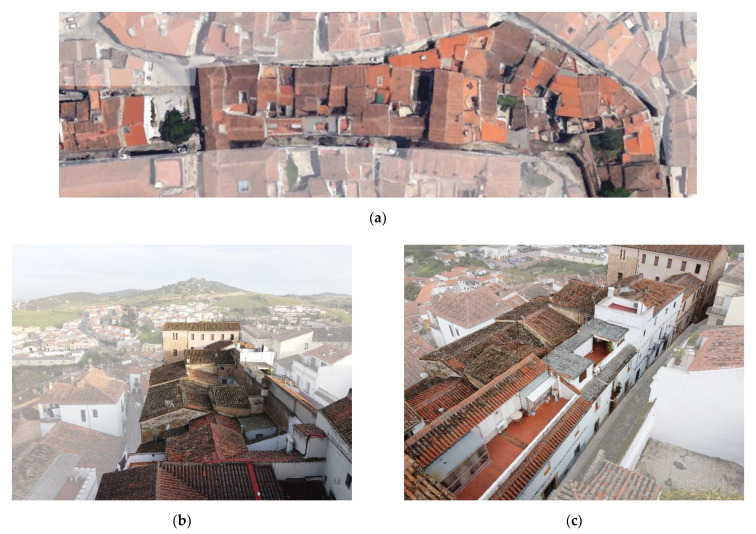
(**a**) Detail of the study area in which the residential growth where the hypothetical military remains can be seen. (**b**) Detail of the inaccessible roof area. (**c**) Detail of the hypothetical intrados urban growth.

The way in which we understand our historic centers today is subject to constant changes derived from the tools [1,2,3] we use to interpret them, especially digital tools and data acquisition and processing procedures. These new digital tools are expanding our capacities to study the city and increasing our research scope. Not long ago, our studies were limited to a building’s three-dimensional models, whereas today we can study large urban areas developing urban scale parametric models (Figure 1).

The knowledge of today’s urban historical and cultural contexts is determined by the constant interpretation systems evolution, thanks to the continuous development of digital tools and data acquisition and processing. At the same time, as mentioned before, the scale has changed; today we can create urban models that combine different techniques [4] such as: traditional photogrammetry, Unmanned Aerial Vehicle (UAV) photogrammetry, terrestrial laser scanning (TLS) and LIDAR models. These models, once combined through different techniques [5], can generate a three-dimensional one with different layers of information. This model allows a highly precise analysis of the urban environment and, more importantly, a non-invasive tool to document and preserve our cities with an overall vision [6]. However, these models demand tools and methodologies that simplify interoperability among them to facilitate the researcher’s work [7].

In order to develop a methodology that allows us to encode, interpret and finally manage the historic centers from the complexity of the urban cells, the research presented in this work develops the study case of a highly reliable and relative easily developed parametric model of an urban area of UNESCO’s World Heritage City of Cáceres. This urban area was selected because it is a historic military zone where, until the date of presentation of this research, the XII century’s Almohad was considered to have been demolished to allow the city’s urban growth. The research team had the intuition that hidden and unknown fragments of the Wall had remained until today.

Thus, this research had a double mission; firstly, to develop a parametric model to improve our knowledge of the historic centers from an overall view and secondly, to locate a series of lost fragments of the Almohad Wall that would serve as practical case to test the validity of the digital methodology presented [8,9].

The works on this area were carried out in 2019. Two research campaigns were carried out. Each campaign had different degrees of intensity: the greater the area studied, the less detail. The first campaign covered an area of more than 7578 m^2^ and in the second campaign the study area was reduced to 1798.94 m^2^ to allow a more detailed and accurate study.

**Figure 2:**
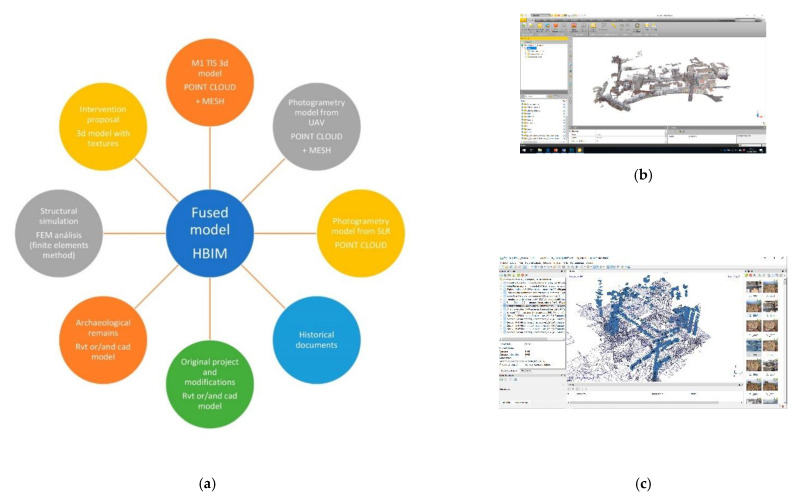
(**a**) The key to this project is the use of a Historic Building Information Model (HBIM) tool that serves as an articulating element of the different databases obtained, guaranteeing their interoperability with a high level of reliability. (**b**) Terrestrial laser scanner (TLS) model, processed in Real Work (**c**) Work in progress of the Structure from Motion (SfM) model in Agisoft Metashape.

These campaigns developed a rapid digital reconstruction of the study areas with parametric 3d models characterized with different information (Figure 2a). The activities focused on the definition, testing and development of a methodology based on easily replicable procedures applicable at this work’s scale and at major and minor scales. The workflow developed was aimed at optimizing the best use of each instrument: LIDAR, UAV and reflex cameras (Figure 2b,c), for its integration into a final and replicable model [10,11].

## 2. Related Works

In the field of heritage and more specifically in the use of BIM (Building Modelling Information) tools to parameterize reality, there are many researchers who have worked on workflows that systematize communication between the capture of reality and modeling tools.

The incorporation of UAV systems in combination with SfM (Structure from Motion) methodologies has generated an immense capacity to study our buildings and historical sites as shown in recent studies [12,13,14]. What began as study tools for buildings and archaeological sites [15,16] nowadays has extended to historical urban areas, thanks to the equipment’s improvements (better and lighter cameras and better computers with more computing capacity) and to the powerful SfM process software tools in the field of analysis. Today, not only are we capable of analyzing a building but also a city.

Currently, it is necessary to integrate those models obtained by photogrammetry (ground or aerial) and the point clouds obtained through TLS within the BIM workflow. This integration and its implementation within real projects with real results is opening a path of possibilities that we could not imagine a few years ago.

The state of art’s analysis shows us different works of great interest that are currently being developed and that open a world of vast possibilities when it comes to study our heritage. Among these ones must be highlighted the advances that are being carried out in the complex surfaces’ modeling from point clouds using Rhinoceros and the modeling automation of some elements through Grasshopper [17]. These advances allow us to model elements with qualities and physical properties (thickness, deterioration, arrows, turns, etc.) similar to construction ones before industrialization (it is evident that a historical construction is characterized by lacking orthogonality) The use of tools such as rhinoceros allows us to achieve a precision’s high degree when obtaining meshed surfaces from reality captures. At present, this workflow has also been improved by Rhinocerus with Rhino Inside REVIT (Rhino-Inside technology allows us to run Rhinoceros like any other plug-in within Revit and AutoCAD), which opens a range of possibilities that must be analyzed in depth.

Nevertheless, there exists a traditional modeling that relies on three-dimensional models obtained by UAV and TLS to recreate buildings. In this modeling we obviously have to do a processing/study of the information that comes to us in which we synthesize it, mainly the constructive element’s geometry, to obtain a cleaner and more operational model [18]. These cleaner construction elements can in turn be exported to other calculation programs. In short, two different models, one more faithful to reality and the other more agile or with more interoperability capabilities.

We can extend the scope of the previous model by adding new layers of information. In this way, as temporal phases, layers of information based (or formed from) on historical documentation can coexist, such as historical photography, historical drawings or even descriptions that help us to model disappeared parts [13,19,20,21].

All the previous models, and without doubts the parametric modeling and the new ways of understanding the architecture’s drawing, open to us an immense range of possibilities to improve and facilitate maintenance, conservation and restoration [22]. The possibilities of incorporating information are practically infinite, as we can incorporate almost any value that is necessary to enrich our group and walk towards smart cities [23].

In our case, the proposed workflow was designed to optimize each instrument’s best use (UAV and reflex cameras), for the integration of terrestrial and aerial photogrammetry with TLS point clouds in a HBIM (Historical Building Information Modelling) platform; a platform that optimizes the data’s workflow. On the other hand, it was applied in a real case. The study case consisted of locating a hidden section of a XII century wall. This section was not documented in the historical sources nor cataloged in the current management instruments since it had remained hidden among the constructions that had been attached to it over time.

Previously, the research team had already obtained important results in a nearby area in which it had located the remains of the Almohad Wall that were considered lost [8,15,24]. These remains of the Almohad Wall, which were considered missing and were poorly located in the official planimetry, were located, positioned and preserved through its inclusion in the city’s Preservation Plan. In that case, the study area was relatively small, approximately a study area of 500 urban m^2^, compared to the new study area that covered about 1798.94 urban m^2^ (Figure 1a–c).

## 3. Theoretical Framework: What Are We Looking For?

Architectural elements are subject to a life cycle, thus in the same way that a living being is born, grows and finally dies; an architectural element has a similar evolution between its appearance, its development and finally its disappearance. However, sometimes it happens that this disappearance is not real, but through processes of concealment, what was considered as eliminated has remained.

**Figure 3:**
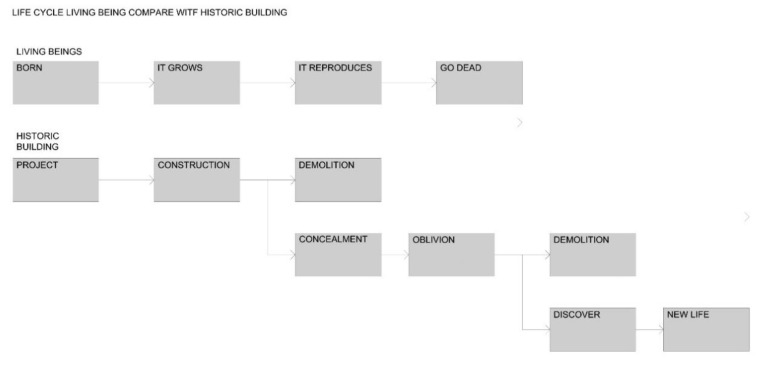
Comparative diagram of the living being life’s cycle of and a historical building showing the possibility of concealment.

This concealment occurs because the documentary sources do not reflect the existence of the element, so it is lost in memory; and because the element is not physically visible or accessible in a direct way, so it is lost in the environment. Finally, the element falls into the category of the disappeared. This situation obviously avoids its valorization and preservation since what it does not exist cannot be cataloged (Figure 3).

This case is especially significant in those architectural elements that have been modifying their function over time, so they have undergone changes to adapt to their new one. The walls are a paradigmatic case, since over the centuries they have evolved from having a delimiting and defensive function of the urban perimeter; to being transformed into dwellings using their walls as parts of these; to being eliminated once they were considered as obstacles to the development of cities. Furthermore, this evolution has not been uniform throughout the whole element, and thus different parts of the perimeter can be found in different stages. Some parts may have already reached a final stage and have disappeared; others may be present, but have developed a residential use, and others, however paradoxical it may be, may be emerging, for example, the historicist rehabilitation of a section of a defensive wall (Figure 1a–c).

In the case of the missing walls, these are generally sections that were physically eliminated by the wall’s demolition processes due to the city’s growth or by its ruin due to wear or natural disasters; but it can also be a hidden heritage. Concealment may have occurred because these sections became part of the village mixing with the urban fabric, and this fact was not documented.

It is in these cases that the application of new technologies can help in a non-invasive way to make comprehensive models of these complex areas; models that allow us to study the village from a new perspective in search of hidden remains of the monument; remains that once located and confirmed by traditional study methods, can be cataloged and thus preserved and valued.

## 4. Developed Methodology

### 4.1. Case Study’s Definition: Northeast Section of the Cáceres’ Wall

The use of a TLS System was justified by the need to generate an agile procedure that helped us to read the study area’s urban structure and that could be extrapolated to other city areas. Since these point clouds were referred to an arbitrary and unscaled system, we used the partial model obtained by means of TLS to register the different models in a complex system. This system was referred to an absolute coordinate system using control points that allowed applying the corresponding transformation (scaling, rotation, and translation in the three axes) [25]. Moreover, this partial model, obtained through TLS, also helped us to validate the precision of the clouds obtained through SfM.

**Figure 4:**
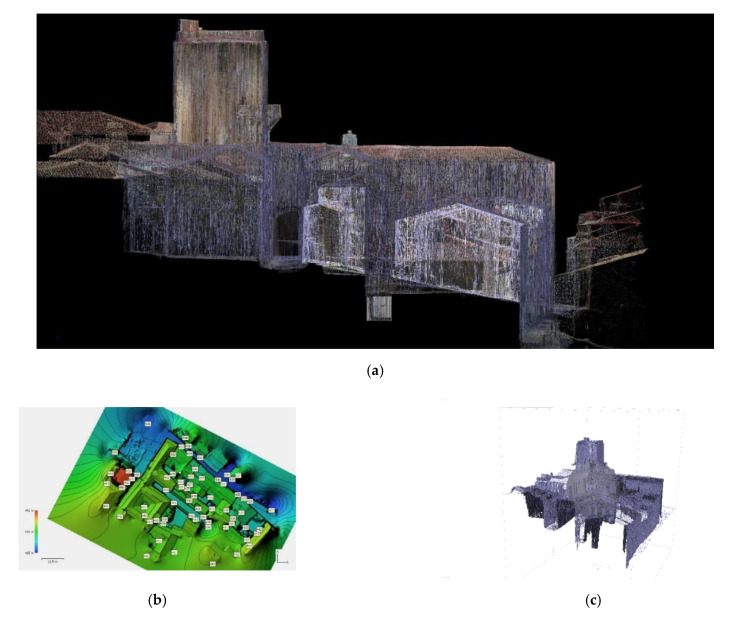
(**a**) Image obtained from the fusion of the 2.5D colored mesh and wire mesh models. (**b**) Digital elevation model obtained through a 2.5D procedure from SfM model (**c**) Sectioned perspective of the polygonal mesh obtained through the 2.5D procedure.

Once the point clouds’ quality was verified and these were approved, the next step was to obtain a high-precision topographic digital elevation model (DEM). This model was made with all the information collected and allowed the research team to discretize between natural topographic barriers and manmade ones using three-dimensional and two-dimensional documentation (contour lines) (Figure 4a–c).

The works consisted of two campaigns as described in the next section. The first campaign (see Figure 4a,b) covered a 7578 m^2^ urban area. The second campaign worked on a 1798.94 m ^2^urban area affecting a total of 14 buildings of different types and construction systems. The second campaign’s area was reduced for operational purposes. In the second campaign, the study area was densely constructed with few courtyards and empty spaces. The total area of courtyards was 257.87 m^2^, 14.33% of the total, as shown in the following table (Table 1).

This area consisted of a heterogeneous set of residential buildings asymmetrically arranged. The set was limited by three streets: Obra Pía de Roco Street, Adarve del Cristo Street and Hornillos Street. Regarding the interior the study area, we started from the premise that there were interior patios that, depending on their location and disposition, could offer us some information about the lost wall. However, we did not have any topographic plans that showed what happened inside the area such as the buildings’ sections, how these rested on the ground or the streets’ configuration.

Cacere’s old city residential construction is characterized by the reuse of previous constructions to save means and economize [24]. This fact has created a very interesting residential architecture with vaulted houses that combine different structures from different times [26] including the military structures among them the XII century Almohad Wall or the 19th century adobe walls. The SfM acquisition was aimed to optimize the movement of the DSLR (digital single lens reflex) camera to generate chunks of high-density points and integrity of each surface [27,28,29].

In order to collect roof data and obtain roof information, in some cases it is essential to use UAVs that allow us to carry out a morphological analysis of the urban landscape, obtaining another second SfM model on a smaller scale [30,31].

It is necessary, as we have already said, to integrate the SfM models obtained both by SLR and UAV cameras with a TLS (terrestrial laser scanner) model to be able to verify the confidence in our point cloud, assess deformations, scale losses. It should be noted that in many areas we will access areas where TLS systems have not been able to access, which is why they are complementary systems.

**Figure 5:**
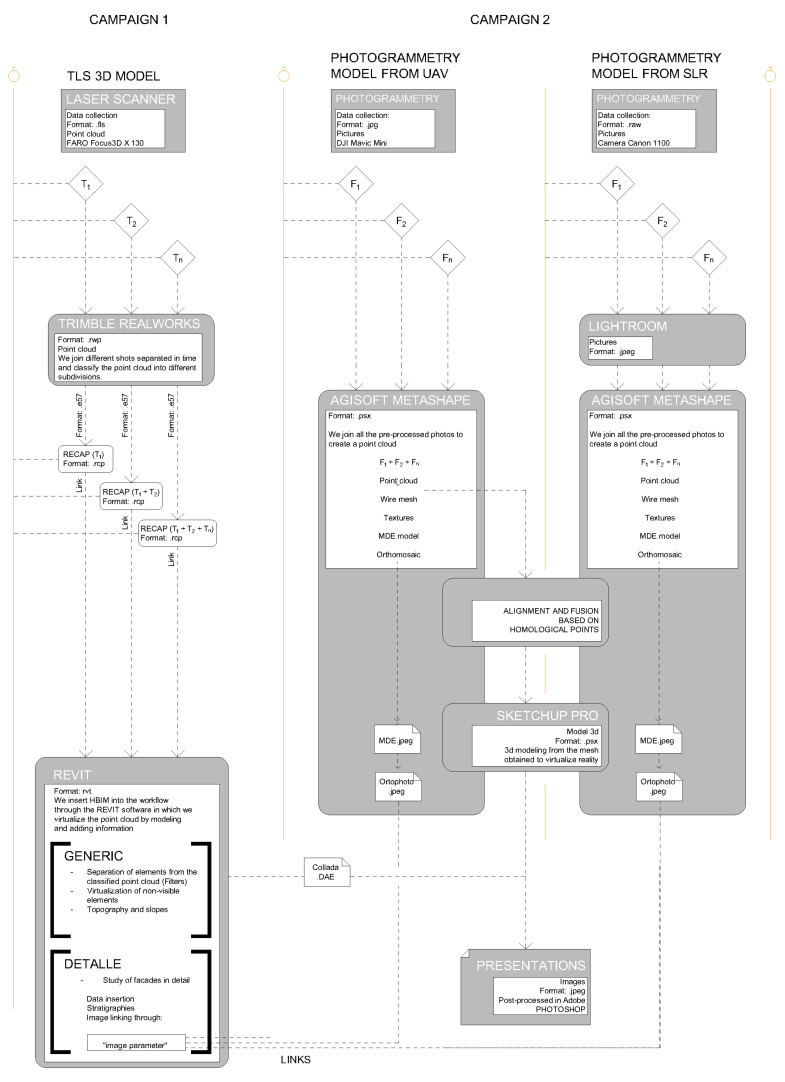
Workflow 1: Cabinet and field works for the first generation of 3D models using SfM and TLS prior to incorporation into HBIM. First results through presentations.

Finally, in an HBIM model, we will have a platform that combines different parametric models, serving as a hub to work with point clouds/obtained through TLS, SfM, meshes, textures, etc.) [32] and thus have reached our management and control tool [6] (See Figure 5).

### 4.2. Planning Works: 1st Campaign TLS Acquisition and 2nd Campaign Aerial and Terrestrial Image Acquisition

#### 4.2.1. Campaign: TLS System

**Figure 6:**
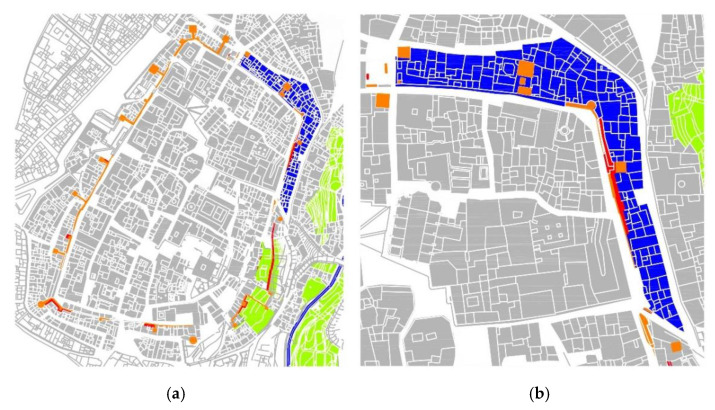
(**a**) Blue: area of action compared to the intramural area of Cáceres. Orange: remains of the Almohad Wall (**b**) Detail of the area of action in blue and of the supposed conserved remains known before the results of the present investigation.

The objective of the first campaign (see Figure 6a,b) was to generate a first closed polygonal model of the study area based on a high-precision point cloud taken with a focus X130 laser scanner. In this campaign, 107 scans were performed. The entire set of scans added up to a total of approximately 182 million points.

The polygonal survey consisted of scans approximately 30 m apart. This procedure attempted to achieve two objectives. The first one was to obtain information about the ground and the vertical elements, which in some cases were house facades and in other were wall elements or defensive towers. The second one was to mark the location of the targets using geopositioned steel tips to allow us to retake and expand these scans in the future and reference them into the main model; so, if in the future we had the possibility of accessing a property, we could go back to the physical mesh of steel spikes placed in the pavement to continue expanding our model obtained through TLS [27,33].

**Figure 7:**
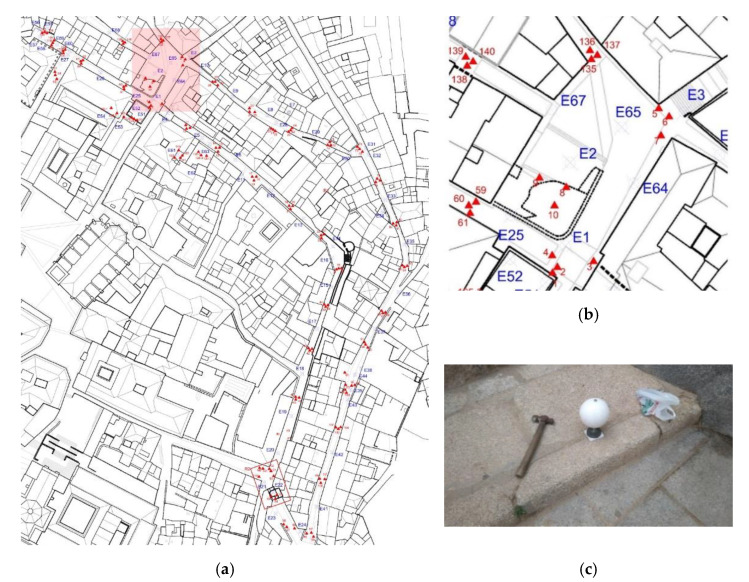
(**a**) Plan of the mesh established for the placement of the spheres for the TLS (**b**) Detail of target concentration areas to ensure their visibility in subsequent campaigns. (**c**) Placement of one of the spheres using a topographic nail.

The procedure followed within the research project [15] for data collection was based on a high-density sphere mesh separated by a maximum of 30 m (see Figure 7a,b).

The position of each sphere within the mesh was geolocated on the pavement using easily locatable topographic marking nails. All these nails were clearly and concisely positioned in a GPS (Global Positioning System) with centimeter precision locating each element in absolute UTM (Universal Transverse Mercator) coordinates (see Figure 7c).

This high-density mesh was essential for the research project as we understood the models under elaboration as living models. Regarding living models, on one hand, because the city is alive and grows responding to new needs, we may need to expand them to reflect these changes. On the other hand, because the models were not complete, in future studies they would have to be expanded with the interior of the surrounding houses, as at present the studies had only looked for the set’s geometry.

This dense mesh served as an anchor point between the three-dimensional models. As mentioned, all the typographic mesh points (see Figure 7a,b) were geolocated and shared by all the models, using anchor points to interconnect all the three-dimensional models [34].

This first model obtained was incomplete as it did not have the capacity to obtain data relating to roofs, courtyards, and interior facades. This model allowed us to screen the study area, reflecting the topography and the height of the buildings. Through this screen we were able to geolocate the wall’s intrados and extrados to know the different heights of the barbican, this was not possible until this moment as this urban area was occupied with residential buildings that avoided a clear view of the area.

**Figure 8:**
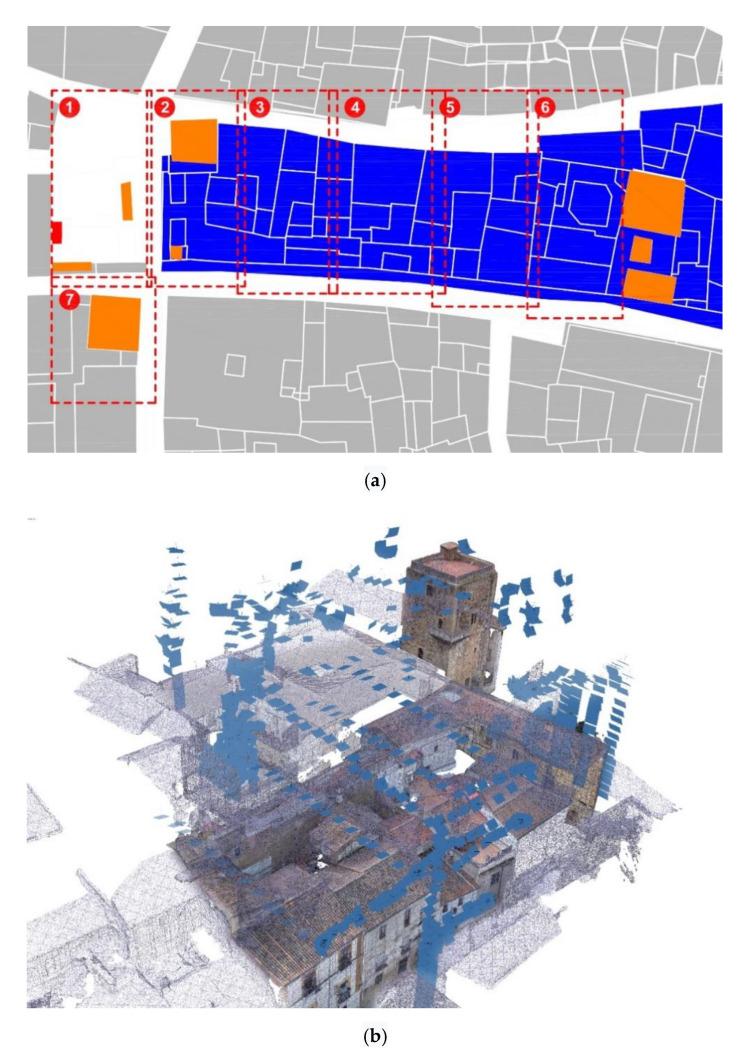
(**a**) 2nd campaign study area, each quadrant divides the 7 surveying missions carried out. Orange: known remains of the XII century wall; blue: 19th century residential growth that have absorved military constructions. (**b**) Fused model of the triangulated mesh and the textured mesh in which the methodology followed in the different missions when carrying out the flights can be appreciated.

Once this area was analyzed (Figure 6a,b), we proceed to define what it would be the 2nd campaign carried out by means of a UAV team and an SLR (Single Lens Reflex) team (Figure 8a,b).

#### 4.2.2. Campaign: Aerial and Terrestrial Image Acquisition

The great distance between the two streets that limited the study area, the morphological diversity of the residential units that composed it, and the impossibility of accessing certain areas (unoccupied houses, roofs, etc.) led to the inevitable choice of an acquisition program Image by UAV complementary to images acquired by terrestrial photography.

This campaign took place in the first half of 2020 (months of January and February) with a small Dji Mavic Mini team weighing less than 250 g, as a non-profit or commercial informative activity and outside the birds’ breeding period (April-May to September-October). As it was a flight with a leisure drone without a combustion engine and the area was part of the Natura 2000 European Ecological Network, we complied with Decree 110/2015, of May 19th, in its Annex I, and that did not require an affection report.

This Dji Mavic Mini was the UAV used to create the point cloud of the entire study area delimited in the second campaign. For this, this second study area had been divided into 8 study areas, each corresponding to one flight mission of the UAV team, which was limited by flight time, weather conditions, operator visibility.

These flights were integrated with terrestrial photogrammetry with an SLR camera with the objective to generate a parametric HBIM model that combined the different models: the one taken with UAV and SLR and the one taken with TLS in order to assess the quality of the different point clouds and their geometric reliability [14,27,35].

### 4.3. UAVs and Terrestrial Acquisition and Post-Processing: Scan to BIM

The use of photogrammetry and laser scanners to study Heritage is especially interesting as it allows capturing highly complex buildings with a very high degree of precision without the traditional data collection’s limitations [16]. The integration of these techniques in the HBIM workflow is a step forward to guarantee our heritage’s preservation and maintenance and to design interventions on our heritage more respectful with the monument [17,18,19,22].

**Figure 9:**
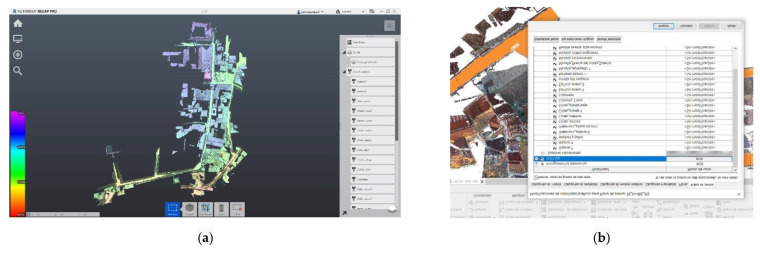
(**a**) Organization of the TLS model according to scan locations that match building elements in RECAP. (**b**) Visibility chart of the different graphics in which we can activate or deactivate the constructive elements as they are organized according to the scans.

The validation and checking model were obtained using TLS Faro Focus, processing the point cloud using the Faro Scene software [24,25]. With this software, an alignment of the different scans was carried out semi-automatically thanks to the spheres described above. We decided to organize the point cloud not based on the scans but based on the construction elements that interested us for the interpretation of the historical set. Accordingly, since we were conducting a non-invasive analysis of the wall, we made groups of residential buildings’ roofs and facades, Roman archaeological remains, Almohad archaeological remains, and reconstructions, as shown in Figure 9a.

The minimum precision values to validate the TLS model and that this later serve us to check the photogrammetric models were between 2 mm and 4.00 mm. This level of precision was not guaranteed either on the roofs or in the areas close to them where we had much larger deviations. All artifacts were removed from the model to improve its understanding. The project, as already indicated above, had some anchor points that had been georeferenced and to help us to link the different models. These control points were those described above and that made up the work mesh. In this point cloud, a resample of the point cloud was carried out prior to export. Once this TLS model was validated, it was exported in * LAZ format (file format containing point clouds) and imported into RECAP(intellingent software for 3d modelling) to prepare the cloud for import into REVIT (Figure 9b).

The photogrammetric UAV model was developed with the Agisoft Metshape 1.6.2 software to generate a dense point cloud for each of the seven missions. Each of the missions was treated as an independent work block [27] for which sparse cloud, dense cloud, mesh, and a digital model of DEM elevations were obtained. Subsequently, the models of the seven missions were aligned, obtaining a general model composed of 1744 cameras that resulted in a dense point cloud of 75,676,246 points and 6,135,248 polygonal faces. The point clouds obtained in each of the missions were aligned with homologous points identified in the streets. Note the versatility of this new tool as it allows us to reduce noise and artifacts from the models.

From this photogrammetric processing we obtained two data outputs to incorporate into our parametric model. The first one was a point cloud whose preparation process to insert it in REVIT was similar to the procedure followed with the TLS model, but in this case, Agisoft provided us the tools necessary to classify the points, clean the model, and resample it. Before exporting this model to REVIT, it was necessary to make a previous step through RECAP in the same way as we did in the TLS model.

The second data output was a three-dimensional mesh model with textures. This model in * DAE, * OBJ or * 3DS format was incorporated into our parametric model (note that in the case of REVIT, unless we create families with Formit software, also from Autodesk, they will not be imported). To incorporate this model in REVIT, a previous step was carried out in the free distribution Blender software to allow us to export an * ifc file (note that although derived from the tests carried out, this step could also be carried out in the Sketch up software). Once we made that export, we incorporated it into our parametric model (note that in the case of wanting to use the obtained textures to elaborate photorealistic images we will use the * DAE files, in our case, we used Lumion, but we could also have used Rhinoceros that requires installing different rendering engines like Enscape).

The photogrammetric model from the terrestrial data collection was elaborated with photographs in raw format to maintain the highest quality and integrity in the starting documentation. This documentation was processed in Agisoft Metashape obtaining the same three-dimensional models obtained in aerial photogrammetry with the limitation that there are large blank areas (ghost zones) and the distortions in the data collection when the photographs had not been taken orthogonally [27]. The missions carried out to obtain the terrestrial photogrammetric model did not overlap with the missions carried out to obtain the aerial model. The same methodology was followed as with the TLS system, but in this case overlapping both campaigns in order to have the same reference points and thus obtain time [33].

Note that taking data in RAW (it is a format to store images without compression) format instead of jpg format (see Table 2) in both aerial and terrestrial photogrammetry is recommended [36,37,38]. We will have a considerable increase in the size of the files when obtaining them from the raw camera but during the processing we will be able to improve different aspects of the photography that will improve our results especially in low light situations. Among the modifiable parameters will be the following: white balance, exposure, contrast, highlights and shadows, intensity, saturation and focus. All these values will allow us to improve our data collection in the cabinet if we have lighting problems, improving our results.

Finally, another meshed model was obtained, especially interesting for our study, a digital elevation model. This model allowed us to establish the altimetric elevation of the existing interior courtyards in the residential units and as a result allowed us to develop a theory of the possible primitive topography of the wall and its barbican in a non-invasive way using a 2.5D triangulation.

### 4.4. Validation Process of the Different Outputs: Determine Reliability

One of the key points in this process was the geometric validation of the different point clouds and models. The reality is that for the proposed work, data collection by UAS (Unmanned Aerial System) is transformed into an agile and versatile system that guarantees a very complete model by lacking ghost zones due to its ability to reach almost all points. However, the reality is that the three-dimensional model obtained through software processing may contain geometric inaccuracies that must be checked, which is why this high-precision model obtained through TLS has been proposed, since we know its millimeter precision, which can validate our other models, serving as control and validation of results [5,27].

The process consisted of four steps, in essence, each of the systems used (TLS, terrestrial photogrammetry, and aerial photogrammetry) provided us with its own three-dimensional model with its strengths and weaknesses. The TLS system guaranteed us a point cloud of centimeter quality and an agile tool to expand the three-dimensional model in future missions as long as we kept our anchor points on the study area’s ground. The photogrammetric models were characterized by being a low-cost system that needed a validation process to guarantee that the data collection was correct. The terrestrial photogrammetry was more precise but had more ghost zones. Finally, the aerial photogrammetry was less precise in principle, but reduced the number of ghost zones to almost 0.

**Figure 10:**
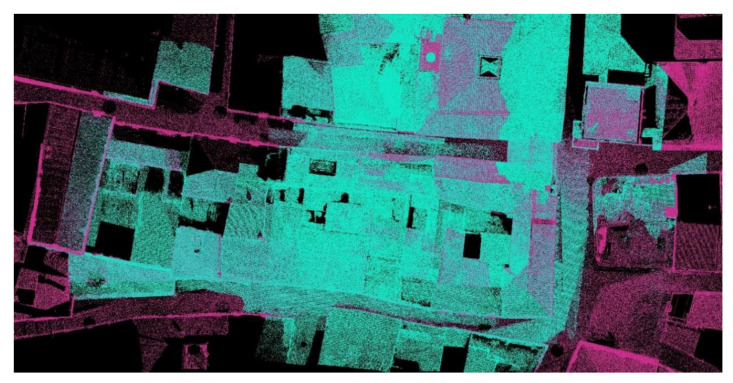
Area view of the two-point clouds to determine the reliability of the models (UAV model and TLS model).

**Figure 11:**
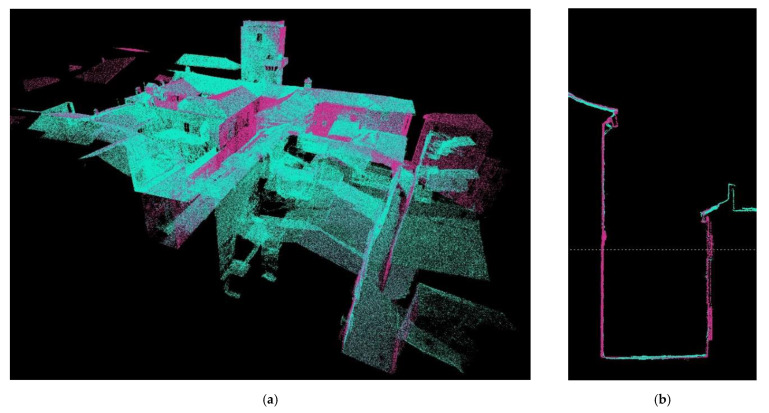
(**a**) Perspective of the fused model in which we can see the limitation of data collection with the TLS system as its vision is limited (blue: TLS model overlap with the UAV model (purple)) (**b**) One of the check sections in which the reliability of the point cloud taken by aerial photogrammetry can be estimated by comparing it with the data collection using TLS, the precision of which is known (centimeter). Blue: TLS model overlap with the UAV model (purple).

**Figure 12:**
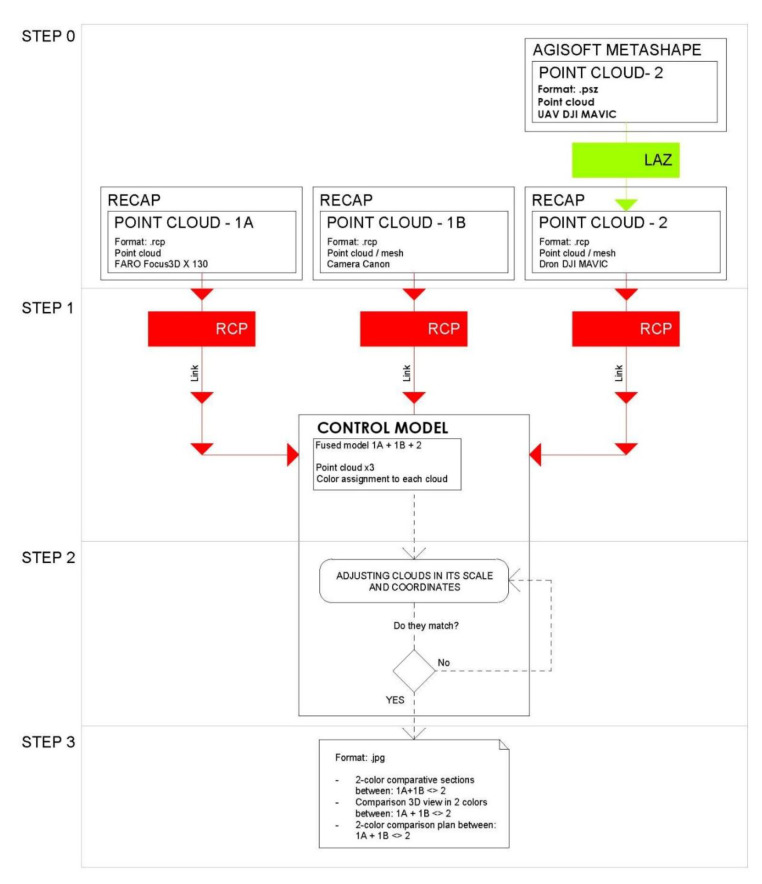
Validation process of the different geometries obtained in four steps.

As mentioned, in this procedure we obtained the different clouds to scale and orient them in our case in the RECAP (Figure 10 and Figure 11) platform to facilitate and guarantee the passage to a BIM (building information modeling), in our case in REVIT platform, as they both belonged to the set of Autodesk tools where interoperability was guaranteed (Figure 12).

Note that when we have the point cloud in other formats, such as psx from Agisoft Metashape, an intermediate step is necessary, which consists of transforming that cloud into a format suitable for Recap. For this, we export from psx to laz. The laz format allows us to load that cloud in Recap, where we can later link it to REVIT. Step 2: when we are in REVIT platform, if we already have the cloud in Recap format, we can link it directly in REVIT as a point cloud that we will call 1 A. We can have as many as we want, 1 A, 1 B, 1 C, etc. The union of the point clouds will be carried out by means of homologous points. Once all the clouds have been fitted in the same position, we proceed to a chromatic differentiation to easily visualize the geometric differences. To do this, we assign each cloud a color, and through volume cuts, 2D sections, plants, we check the different geometries. From REVIT, the handling and superposition of the different point clouds is tremendously agile, being able to activate and deactivate zones to improve the reading of the set and obtain comparative sections of the models in which to check our level of precision [27].

In the previous text we have not referred to the mesh models described above because these are the result of the processing of their corresponding clouds. If the point clouds are validated by extension the mesh models will also be validated. It is not necessary to perform this process on them too. All the steps of the process are summarized in Figure 12.

## 5. Unified HBIM Model

**Figure 13:**
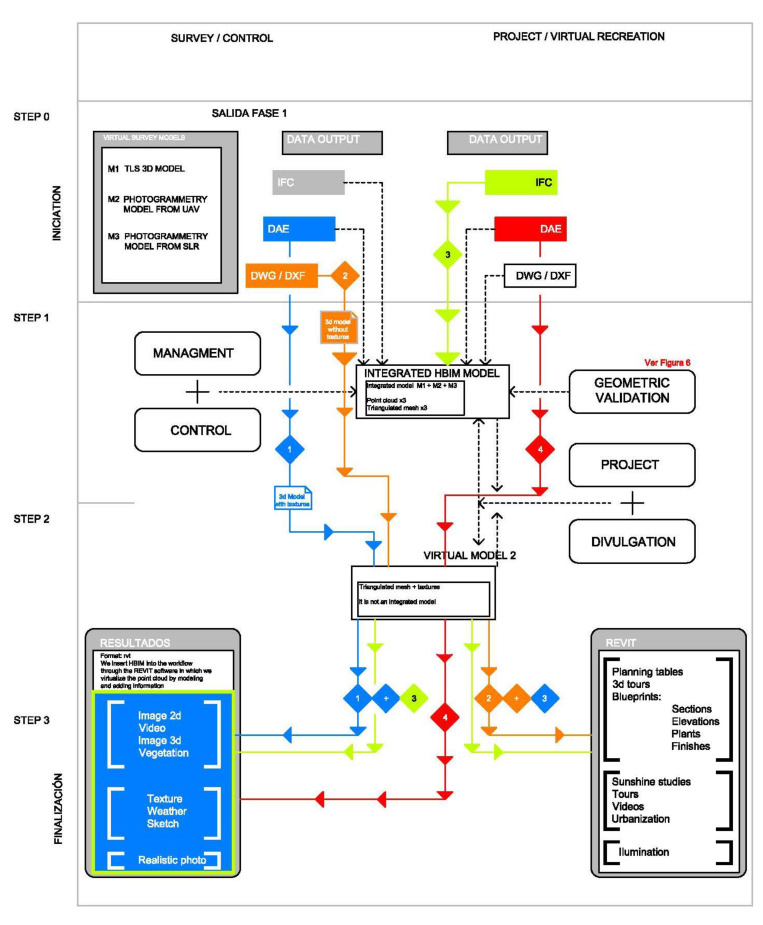
Workflow in three steps and two tours depending on the needs: survey and control and project and virtual re-construction.

Once we validated the geometry of the point cloud, we performed the step reflected in Figure 13. In this step, we had two workflows, each of these flows had a different ap-proach described in Table 3.

A first “work flow 1”: survey, control and project and a second “work flow 2”: control and virtual restitution. These two flows guarantee the maintenance, control, conservation, planning interventions, project and dissemination of our heritage based on parametric bases that can be updated with new information.

The first workflow is developed entirely within the REVIT platform (it is the continuation of the geometric validation carried out in Figure 12) and the result is a central coordination model that combines the obtained models that are described in Table 4. All these models, as indicated in Figure 13, are coordinated in a central model in the REVIT platform, linking them as independent files that can be modified and updated in case the study is expanded to new areas.

The models need to be integrated within a model as if they were disciplines and allow advanced modeling of the whole. Among the advantages of the REVIT platform is its agility for manipulating and moving the overlapping three-dimensional files, being able to use different views depending on the needs. Thus, we can work at all times as if we were in a traditional CAD (Computer Aided Design) program, activating and deactivating the layers according to our needs [15].

This model allows us to perform non-invasive control tasks [6] by integrating the different results of our works (TLS model, SfM photogrammetric models and SfM terrestrial models); all these validated results with a centimeter precision. This workflow allows the BIM administrator/BIM architect to control and inspect the monuments’ geometric and volumetric dimensions, practically without effort guaranteeing the building’s control and maintenance in a completely efficient way.

In addition, these models work with an on and off layer system (see Figure 9a,b), a useful tool for studying buildings and their surroundings. They allow us to eliminate non-invasive city’s overlapping layers that make difficult to read the monument chronologically, but if sequenced, they provide invaluable information about our heritage, allowing non-invasive readings not done till now. These readings are necessary for the study but also for the dissemination because with the new virtual reality technologies we can make visits of almost any type (inspection, study, dissemination, etc.) [6].

**Figure 14:**
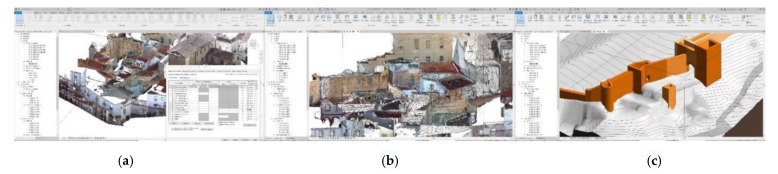
(**a**) TLS model in REVIT with gosht zones (**b**) TLS model + 3d Mesh without textures completing gosht zones (**c**) Virtual reconstruction of the wall.

The approach adopted for the modeling was to establish a workspace in which we could visualize the wall’s remains undoubtedly preserved. This way, modeling from a distant scale to a close one [18,39,40], the military elements that were preserved in the cloud points were prioritized and discretized to allow making hypothesis about possible layouts. Approaching us from a far scale to a close one is an orderly methodology that helps us make decisions and in study these urban groups or urban aggregates [41]. It is important to follow a methodology of this type because in making decisions we will only focus on one aspect of the monument; in the first instance we can analyze the trace to later see the thicknesses or changes in thickness; see Figure 14a–c.

In a historical building it is common both that there is no orthogonality and that there are considerable changes in thickness in the same element. Both features hamper the REVIT workflow. In our model and given that our objective was to know the true geometry of the wall, we ignored the issue of orthogonality, obviously the position of an element is what it is and that is a priority. On the other hand, in the modeling of the missing elements and the assumptions, we did not prioritize the thickness, having a tolerance range in which we admitted deviations of +/− 5%; see Figure 14c [40].

**Figure 15:**
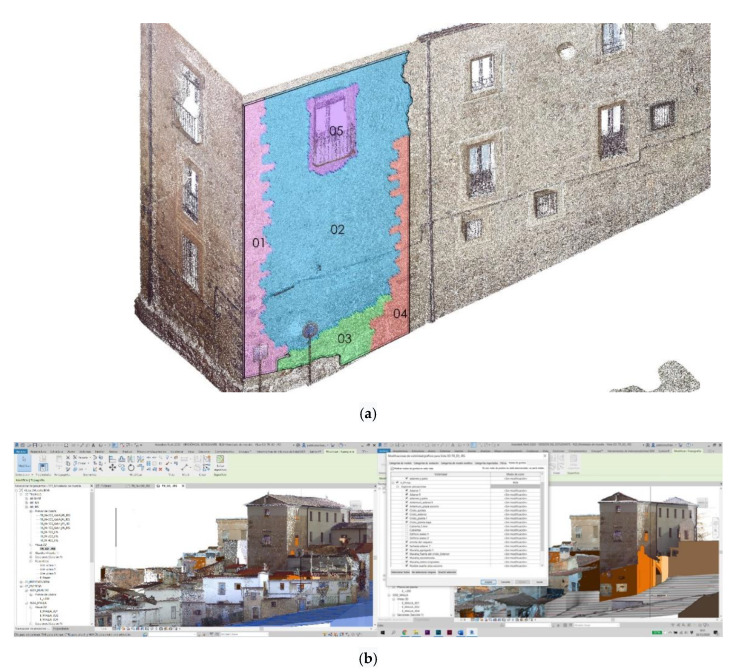
(**a**) Example of a stratigraphic map superimposed on a parametric model on a wall tower (**b**) On the left, parametric model merged with all layers activated. On the right, non-invasive inspection of the interior walls once the external layers have been deactivated.

This model is a clearly versatile model on which you take measurements. On the other hand, being parametric, the incorporation of information is always guaranteed, being able to have a living document with many levels of information, from archaeological layers, materials, finishes, sanitation networks, plumbing, etc.; see Figure 15a,b.

Note that in this second workflow we will prepare the models to be used either in augmented reality or in virtual reality. We highlight the importance in this case of the mesh models with texture, as indicated in Table 4, because they will guarantee this photorealistic experience [42,43,44].

It is important in this workflow to adapt the number of polygons of our three-dimensional meshes because excessive numbers of polygons will make it impossible to use virtual reality tools. This reduction process in our case has been carried out directly from the Agisoft tool in the same way that we will resample the point clouds obtained by photogrammetry. There are other tools on the market that allow us to carry out these operations, such as free distribution blender or Rhinoceros [45]. The final objective is to obtain a mesh with few triangles but with a texture with high resolution.

Once we optimized our three-dimensional model, we used the Enscape rendering engine installed as a plugin in the Sketch up software. The sketchfab.com platform was used to generate models posted on the network to view our work both as a traditional three-dimensional model, and as augmented reality and virtual reality. Note that in REVIT and in Rhinoceros, we can generate virtual reality spaces from our work.

Both these three-dimensional tours in augmented reality and virtual reality as traditional renders in engines such as Vray, Endscape or the Lumion software allow us a hitherto unknown disclosure of heritage; Figure 15a,b.

Currently, it can be combined with calculation programs by having an IFC-type exchange file to guarantee interoperability between the different platforms, we can create measurements and join them to our file, and we can even work with video and rendering tools.

The results of this work are compatible with data management advanced techniques and technologies. It is important to keep in mind the ability to provide personalized useful data to each user. In this sense, the conclusions of the project combined with these new technologies such as the KYRA program (Knowledge-based Information Retrieval from Art collections) can be very useful to present in a more friendly and directed way what was discovered in the project [46].

This type of technology also admits new dissemination channels such as App, Web content, etc., that help to promote knowledge and dissemination of Cultural Heritage. It enables dissemination in a format that allows researchers to improve knowledge without having to diminish scientific or research quality.

An example is a multimedia guide that based on the results of this project that has been presented to the scientific community, as part of the KYRA program. This example demonstrates the parameterization’s versatility of this research project data.

## 6. Discussion and Results

### 6.1. Discussion and Results of the Data Collection

**Figure 16:**
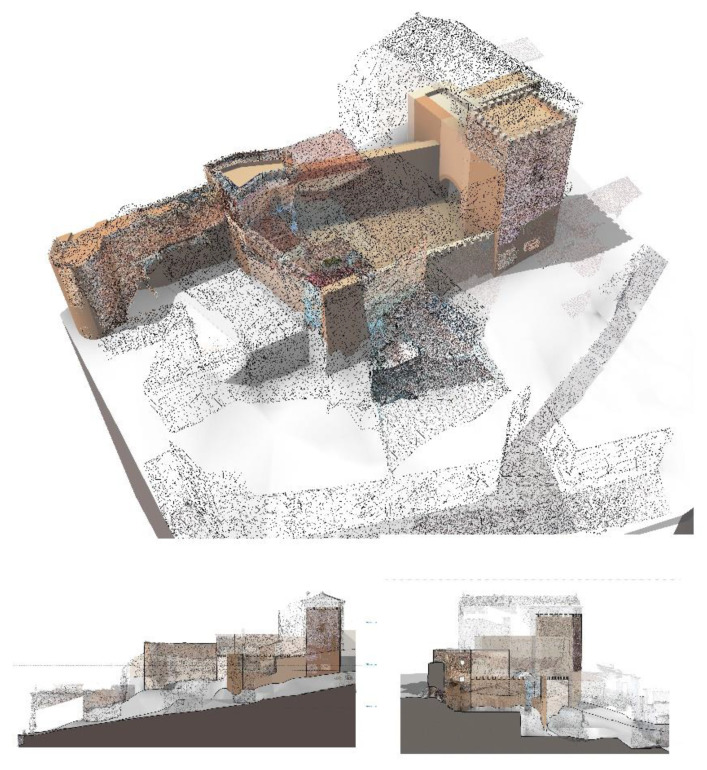
Results for dissemination and study: merged model emphasizing the different stages. Note how the mesh obtained by SfM eliminates ghost areas.

**Figure 17:**
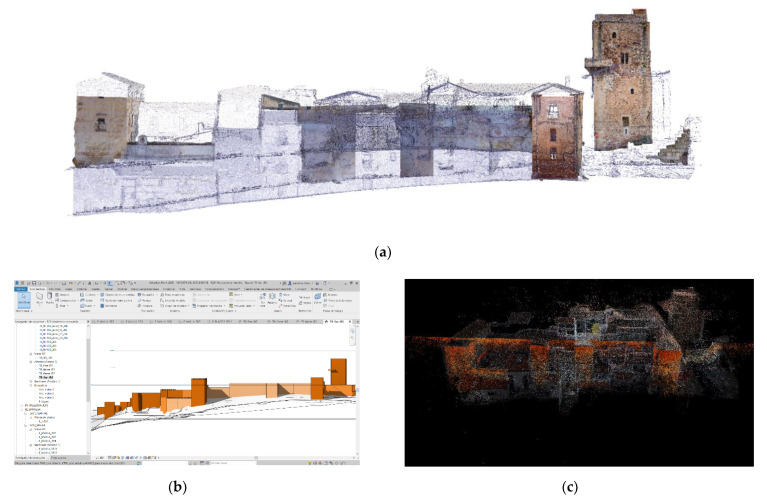
(**a**) Integral model playing with different textures and opacities of the materials to improve the understanding of the layers. (**b**) Virtualization modeled from the different three-dimensional models (**c**) Artistic vision of the whole merging the different layers with different degrees of opacity to improve the understanding of the whole.

From the point of view of the methodology the results for data collection revealed that in a scenario in which we need a low-cost system to capture a constructed reality, we could utilize a data collection system based solely on aerial UAV systems, defining control points to validate the cloud point’s quality obtained. A high number of geometric validation points could even allow us to avoid using TLS systems, thus reducing the campaigns’ data collection costs, although if the economic means and the importance of the study area allow it, the use of both systems is highly recommended (Figure 16 and Figure 17).

On the contrary, the incorporation of UAV systems in combination with SfM methodologies and the substantial improvement in the quality of cameras, lenses and stabilization systems made the three-dimensional model obtained by acquiring ground images practically unnecessary. In the SLR model we can obtain high-quality results thanks to the high-resolution cameras that we have at our disposal as researchers, although we will always be limited to work at ground level, producing three-dimensional models with large phantom areas (roofs, interior facades, etc.). Also, data collection through UAV systems will always be limited by the sectoral regulations that may limit flights depending on the cases. 

The proposed workflows (Table 3 and Figure 13) are an important step in shaping historic SMART cities [42,43,44,45]. Synthetically both workflows cannot be understood separately, both allow us to protect our Heritage in its broadest dimension. As we have seen from maintenance, to control, rehabilitation, and enlargement, etc., new measurement techniques, especially photogrammetric methods compared to TLS technology, allow rapid acquisition of reliable data. The article presents how to use different photogrammetry techniques and the verification of the quality of the information through TLS shots (Figure 10 and Figure 11).

The conversion and import procedure of the different data bases it still requires several software packages for the processing. In our work we have incorporated the different databases in the parametric model elaborated in REVIT, implementing all the different data sets, points clouds, three-dimensional meshes, textured meshes (Figure 9 and Figure 14).

This HBIM model allows us to see in real time the different models (mesh, points, construction phases, elements demolished or to be demolished) creating a powerful asset management tool that can serve all stakeholders [34,47] (Table 4).

Once the different meshes have been superimposed, we have found an efficient and fast tool to be able to analyze our Heritage.

On the other hand, with these low-cost shots, we have a very interesting tool to disseminate our heritage. It is no longer necessary to be on the site to see a monument. The new techniques of total immersion such as VR (Virtual Reality) or AR (Augmented Reality) allow us to democratize knowledge by making t3d models available to anyone with an internet connection. From simple models that allow us to know the building to the incorporation of other information such as texts, planimetry, current or historical photographs to more complex models with augmented reality or even total immersions thanks to virtual reality and the use of more expensive equipment such as Oculus Rift or HTC lives (both virtual are virtual reality glasses).

### 6.2. Discussion and Results of the Analysis of the Case Study

From the point of view of our case study:

Firstly, hidden geometries were revealed showing an unknown urban layout not documented in the existing planimetry. This geometric evidence showed architectural remains prior to the residential development. These remains, due to its geometry and dimensions, corresponded to a military architecture, probably the old wall that supposedly had been demolished.

**Figure 18:**
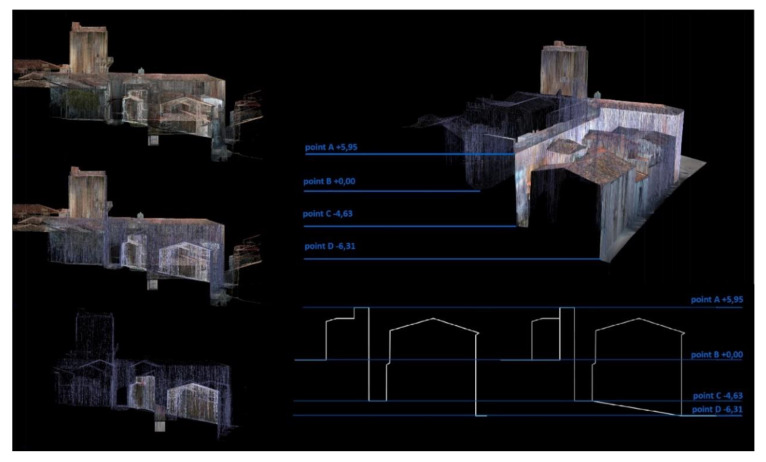
Altimetric analysis of the study area from the 2.5D model.

Secondly, once our 2.5D model was elaborated, we encountered two situations in the courtyards belonging to the dwellings studied. The first one was that the courtyards were aligned with that hidden geometry that we had located in the previous point (see Figure 18). The second one was that the patios marked a topographic unevenness of dimensions that would only make sense if they had been executed for military purposes. Note that there was no realistic planimetry of the study area to know the different elevations in the area prior to the study. (Figure 18).

Therefore, contrary to what it was reflected in the City Master Plan the Almohad wall had not been demolished, as shown in Figure 18. The wall still existed; however, it was not located where it was assumed as it was displaced between 5 and 6 m. The wall, instead of being demolished during the residential expansion, was used as a foundation and facing of the new dwellings to save costs and materials.

**Figure 19:**
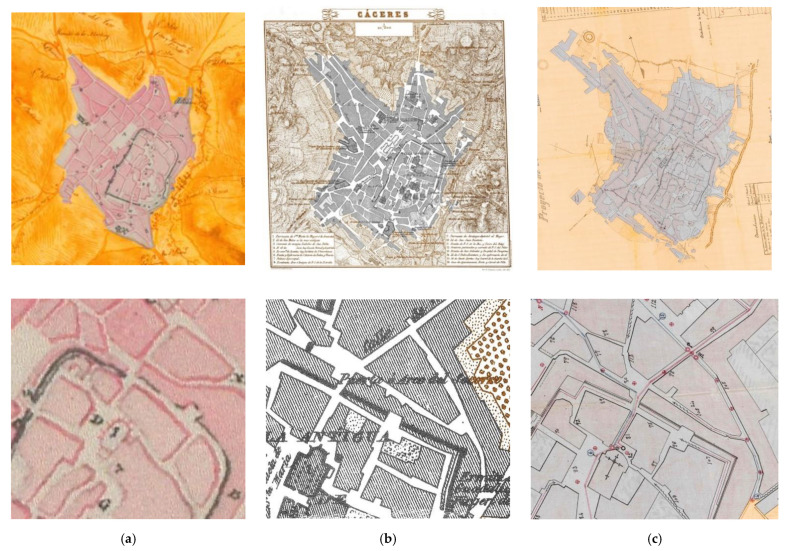
Cartographic analysis. (**a**) Column 1 Baibier’s Plan of 1813 and detail of the study area of (**b**) Column 2 Francisco Coello’s Plan of 1853 and detail of the study area of. (**c**) Water Canalization Project of 1895 and detail.

To confirm the validity of the methodology and check the fact exposed, the research team studied the historical cartographic documentation to check where the wall was in this area in the past (Figure 19). For this, the cartographic resources published by the Cáceres’ City Council were consulted. Surprisingly in three of the documents analyzed, in the study area the Almohad Wall was in the location determined by our methodology and not where the current plans mark it. This location was maintained until the end of the 19th century (the military zone appears graphically for the last time in the water supply plan of 1895 and previously in two other documents of 1813 and 1853 respectively), being displaced in the later representations.

In the first document, Baibier’s Plan drawn in 1813, the wall still existed in this area and its intrados had not yet been occupied by residential dwellings. Also, the two wall sections, the one ending in Obra Pio de Roco and the one starting at Arco del Rey were not aligned, perhaps to create an elbow access.

In the second document, Don Francisco Coello’s Plan drawn in 1853, the wall’s section under study appeared displaced several meters in relation to the actual plans, exactly where this document represents it. Indeed, residential dwellings already appeared in this Plan and instead of being placed on the extrados of the canvas, they used their intrados, which makes a lot of sense because obviously towards the extrados we would have a slope that would make it impossible to use them.

The last document analyzed was The Drinking Water Supply Plan drawn in 1895. In this document, the wall was represented in the middle of the residential dwellings because, in the previous case, the wall was being used by these as part of their facings.

## 7. Conclusions

### 7.1. Conclusions Regarding the Proposed Workflow

The article analyzes a workflow for the generation of three-dimensional models from TLS and aerial and terrestrial photogrammetry, applied in a concrete case of the urban context of the City of Cáceres, a World Heritage Site. The lessons learned and conclusions of this workflow divided into two parts: field work-data collection, and office (obtaining three-dimensional models from the different data collection and validation). Thus, we have the following:

The preliminary analysis of the urban configuration is an operation of paramount importance to program the work missions and thus optimize the data acquisition campaign.

The use of UAVs as a tool for data acquisition becomes a tremendously useful tool that enables us to obtain three-dimensional models unthinkable with traditional tools such as terrestrial photogrammetry and TLS. The use of UAVs guarantees the collection of data from large areas of land quickly and efficiently.

The use of TLS systems to calibrate the three-dimensional models obtained by aerial photogrammetry confirms the reliability of these models. The fusion of both models in a HBIM parametric environment allows us to generate a complex model with different layers of information that guarantee a surprising quality of information.

The models obtained by terrestrial photogrammetry become less effective outdoors, perhaps being relegated to future extensions of the parametric model in which interiors of buildings or areas of poor accessibility and maneuverability are incorporated in which a terrestrial system is operational.

As mentioned, the data acquisition, post-production and verification of the quality of the three-dimensional models obtained by UAV was tested in a particularly complex part of the city, characterized by a dwellings’ agglomeration with different volumes that hided the geometries of previous elements, such as the Almohad Wall. There was a certain uncertainty in the quality of the final database that we could not leave behind, so we had to carry out constant checks that guarantee the quality of the information. For geometric validation, it was essential to have the equipment and work procedures that guaranteed centimeter precision. In our case, the use of the point cloud taken through TLS and the use of the georeferenced mesh with a centimeter GPS allowed the creation of a database suitable for the validation of the three-dimensional models obtained by photogrammetry. In the case of not having TLS equipment, the use of topographic instruments, such as total stations, becomes essential to control the error of the different point clouds. In our case, the verification with the TLS model gave satisfactory results.

The models’ fusion in a final intelligent digital platform that allows us to interact with the different models and serves as the basis for both the control and maintenance of the Heritage and opens research future lines for future projects is the study’s final result. In this new digital era, parametric models in BIM technology allow us to link what was previously unthinkable: a multitude of point clouds, three-dimensional meshes, textured models, etc. This in turn allow us to generate a parametric model in continuous change and evolution; with the capacity of being updated as our society or our ability to incorporate information advances; and of working three-dimensionally with all this information in real time, allowing us to obtain cuts, sections, elevations, topography, etc. On the other hand, the archaeological layers have also been incorporated on the three-dimensional models, obtaining a model with the incorporated stratigraphy [28].

We remark the possibility of incorporating augmented reality and virtual reality technologies thanks to workflow 2. Making our monuments available to the population through these technologies is essential to democratize architecture by facilitating access to sites that until moment were inaccessible [46]. This universalization will guarantee that we all know our past regardless of where we live and the distances, thus access to these databases from schools will allow any child to access a monument from their school with minimal investment.

From an educational point of view, these databases and this new way of interacting will take us a step further on what we know, in the same way that in the 1980s we used paper encyclopedias, in the year 2000 tools such as digital photography appeared, and videos (all of them in 2d) were driven by the democratization of Internet knowledge. In the year 2020, favored by technology, we will access cultural sites through a computer.

**Figure 20:**
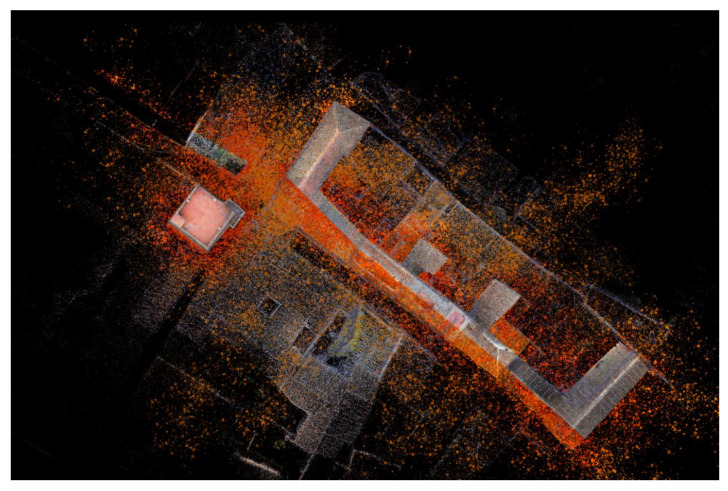
Virtual recreation of the localized remains superimposed on the three-dimensional model.

From a social point of view, these databases will guarantee a universalization of our heritage through new technologies (Figure 20). These first steps in that universalization will generate the possibility of visiting a bell tower or a church not limited by our physical condition or distance. Thus, people with reduced mobility will be able to see what has only been for generations allowed to people without limitations, such as a bell tower [48].

### 7.2. Conclusions Regarding the Findings

First, the wall’s section under study is a genuine fragment of the Almohad Wall that had supposedly disappeared [16,17]. It is a powerful canvas, which according to its dimensions, layout, and location with respect to the orography of the land, is undoubtedly part of the Almohad Wall that defended the City of Cáceres. This section is built with masonry. This masonry may be the substitute for the very degraded adobe concrete walls on this flank, the one most exposed to the attacks of the northern armies in the various sieges that suffered the city during the XII and XIII centuries. In any case, the section can only be seen from the courtyards of the houses as it is integrated into the homes; therefore, an archaeological study in detail is necessary to determine the different construction periods through a stratigraphic study.

Second, the location and position of the wall’s section under study corresponds with what is reflected in the historical cartography of the city. In this planimetry it is reflected that the section in question never disappeared, as it was reused and integrated in the construction of the houses. However, in the current city’s cartography, the wall’s location in this area is misrepresented (Figure 19 and Figure 20).

In the current recreation plans of Caceres’ Almohad Wall, the wall’s section under study is aligned with the front facades of Calle Obra Pía de Roco, whereas this study demonstrates that it is aligned with the rear facades of these houses. This displacement was already demonstrated in Calle Adarve del Cristo after the rehabilitation of house No. 5 and continues in Calle Obra Pía de Roco as it follows the orography of the area. Furthermore, this displacement corresponds to the location of the wall in the historical plans.

**Figure 21:**
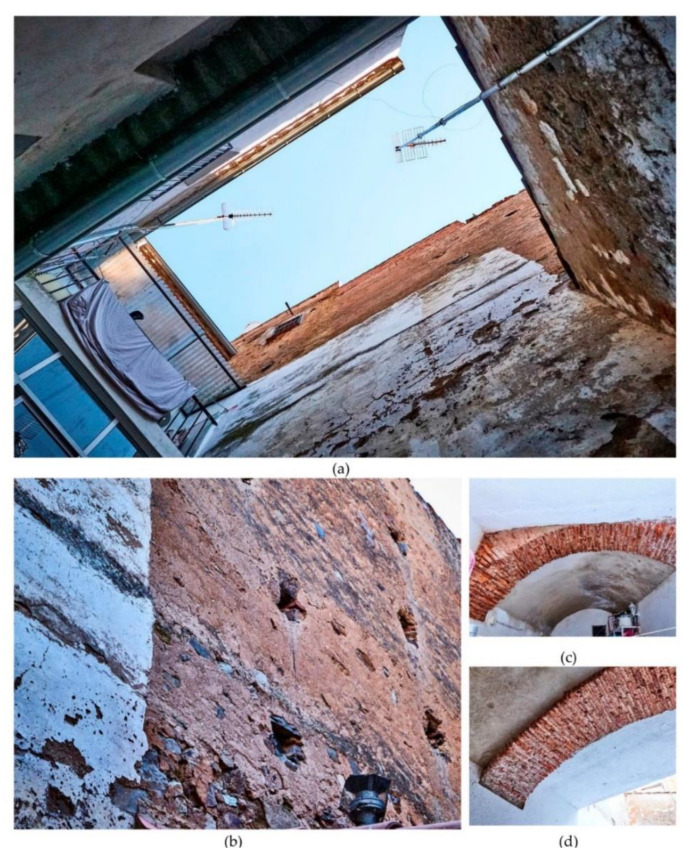
Visual inspection of the discovered walls. (**a**) View of the patios and how the new houses are attached to the pre-existing wall. (**b**) Detail of the old mechinales used to build the wall (**c**) Imposing arch and barrel vault under one of the houses (**d**) Start and detail of an existing arch.

The study of the city’s cartography and documentation leads us to other interesting findings regarding the doors and shutters of the wall in this area. A shutter located on Calle Adarve del Cristo n° 5, named San Miguel’s shutter, and a gate, named Arco de Caleros, reflected in the Francisco Coello’s Plan of 1853; this gate would explain the width of Calle Caleros at the t numbers 42 and 44. These findings are being studied by this research group to complete the research of the wall in this area (Figure 20 and Figure 21).

## Figures and Tables

**Table 1:** Second Campaign’s Research area.

Property		Floor Area	Patio Area	Protection Level
1	Obra Pía de Roco 1	211 m^2^	-	Ambiental singular + integral
2	Obra Pía de Roco 3	67 m^2^	-	Ambiente singular
3	Obra Pía de Roco 5	56 m^2^	-	Ambiental singular
4	Obra Pía de Roco 7	262 m^2^	19.63 m^2^	Ambiental singular + ambiental
7	Adarve del Cristo 1	556 m^2^	172.36. m^2^	Ambiental + VPS private garden singular
8	Calle Hornillos 2	157 m^2^	13 m^2^	Generic
9	Calle Hornillos 4	92 m^2^	16.48 m^2^	Ambiental
10	Calle Hornillos 6	80 m^2^	8.8 m^2^	Ambiental
11	Calle Hornillos 8	153 m^2^	27.6 m^2^	Ambiental
12	Calle Hornillos 10	-	-	Ambiental
13	Calle Hornillos 12	85.62 m^2^	-	Ambiental
14	Calle Hornillos 14	79.32 m^2^	-	Ambiental

**Table 2:** Comparison table between jpg format and raw format RAW.

Format		bpp (Color Depth)	Compressed	Quality Loss	Comments	Size Increase **	Size Increase Percentage ***
1	Raw	16 bit/14 bit/12 bit *	No	No	Higher image quality making the most of the camera’s capture and tonal range. It is its own format, which requires the use of specific programs or plugins depending on the camera used.	Between 19 and 30 Mbytes.	312.5% biggest
2	JPG	8 bit	Yes	Yes	The image is compressed and reduces the image quality. The tonal range of our camera is reduced. It is a standard format recognized by most devices and programs. It is easy to share.	Between 2.5 and 19 Mbytes	-

* It depends on the camera chosen and the level of compression. Each brand has its own raw format and its variations (Canon CR2, Nikon NEF, Sony SR2, Panasonic RAW2, Olympus ORF, Pentax PTX, etc.). ** To calculate the percentage increase in size, a Canon EOS 400D camera with 18 MP Megapixel CMOS has been taken as reference. *** The percentage has been calculated with respect to average values (25 Mbytes for RAW and 8 Mbytes for JPG).

**Table 3:** Workflow after geometric validation.

	Management	Control	Conservation	Planning of Interventions	Project	Divulgation
Workflow 1	x	x	x	x	x	
Workflow 2		x		x		X

**Table 4:** Three-dimensional models incorporated into each parametric model.

	Workflow 1	Workflow 2
Point cloud from TLS	X	
Point cloud from SfM from UAV	X	
Point cloud from SfM from SLR	x	
3d Mesh with out textures	X	x
3d Mesh with texture		x
3d objects	x	x
3d MEP (mechanical electrical and plumbing) objects	x	
